# Clinical efficacy and safety of Chinese herbal medicine for the treatment of patients with early diabetic nephropathy: A protocol for systematic review and meta-analysis

**DOI:** 10.1097/MD.0000000000020678

**Published:** 2020-07-17

**Authors:** Xiaomei Yang, Chenling Hu, Shengju Wang, Qiu Chen

**Affiliations:** a School of Clinical Medicine; b Department of Endocrinology, Hospital of Chengdu University of Traditional Chinese Medicine, Chengdu, China.

**Keywords:** Chinese herbal medicine, Chinese medicine, early diabetic nephropathy, early stage diabetic nephropathy, traditional Chinese medicine

## Abstract

**Background:**

Diabetic nephropathy (DN) is among the common and serious complications of diabetes and is also a major cause of end-stage kidney disease. Early DN is also called diabetic microalbumin period, the main treatment is in the control of blood sugar on the basis of kidney protection and urine lowering protein. There are few effective methods of western medicine treatment, and most of them are accompanied by adverse reactions. But some studies have shown that traditional Chinese medicine has achieved the curative effect and has certain superiority. However, there are few systematic reviews on the treatment of traditional Chinese herbal medicine for early DN currently. Therefore, this study conducted a systematic review of clinical efficacy and safety of Chinese herbal medicine for the treatment of patients with early DN, aim to comprehensively analyze the role of traditional Chinese herbal medicine in the treatment of early DN.

**Methods and analysis:**

The protocol of this systematic review and meta-analysis was registered on the INPLASY website (https://inplasy.com/inplasy-2020-4-0139/) and INPLASY registration number is INPLASY202040139. A systematic literature search will be conducted in 3 English database and 4 Chinese databases with a language limitation of English and Chinese. Search for clinical research literature on Chinese herbal medicine treatment of DN published in domestic and foreign biomedical journals. The time is limited from January 2010 to February 2020. We will investigate heterogeneity across studies and publication bias. To assess the risk of bias and quality of the included studies, we will use the Cochrane Collaboration's ROB tool. According to the relevant standards in the Cochrane Intervention System Evaluation Manual, it will be divided into low risk, high risk, and unclear. We will also use the RevMan 5.3 software and Stata 13.0 software for meta-analysis of the effectiveness and symptom scores of DN proteinuria.

**Ethics and dissemination:**

The ethical considerations are not required because the systematic review is based on published studies. The systematic review and meta-analysis will be published in a peer-reviewed Journal.

## Abstract 2

Supplemental Digital Content is available in the text

## 1 Introduction

Diabetic nephropathy (DN) is the common complications of diabetes and is a major cause of end-stage kidney disease.^[1]^ As the epidemic of diabetes spreads, the number of patients at risk for developing DN are increasing, which occurs in 20% to 40% of all diabetic patients.^[2]^ The main characteristics of DN include proteinuria, decline in glomerular filtration, hypertension, and high risk of cardiovascular morbidity and mortality.^[3]^ Its condition is complicated and the course of the disease is long, which poses a threat to the patient's life and health. As a result, more and more attention has been paid to the treatment of DN, especially early prevention and treatment. The main risk factors besides elevated serum lipids, include smoking habits, and the amount of dietary proteins, hyperglycemia, hypertension, and genetic predisposition.^[4]^ The goal of DN therapy is reduced the progression of kidney damage and controlled the related complications.^[5]^ A large number of studies about early DN had been carried out, and has achieved some progress in the understanding and treatment of early DN. According to Mogensen Stage, DN can be divided into 5 stages: stage 1, high perfusion or kidney hypertrophy; stage 2, normal urinary albumin excretion rate; stage 3, also called early DN, microalbumin appearing in the urine; stage 4, also called clinical or dominant DN, plenty of albumin appearing in the urine; and stage 5, end-stage renal disease (ESRD). To prevent entry into the ESRD phase, therapeutic measures must be adopted in early stages of DN, stage 3 nephropathy.^[6,7]^ Once early DN (stage 3 nephropathy) has developed into phase clinical albuminuria (stage 4 nephropathy), kidney failure is unavoidable and irreversible, requiring renal replacement therapy. Therefore, early treatments in stage 3 DN is the most important defense against kidney failure.^[8]^ Clinical practice guidelines for the prevention of early DN have been traditionally focused on the control of serum glucose, blood pressure and dyslipidemia, and some emphasis on the renin-angiotensin-aldosterone system as a main target for successful therapy.^[9]^ However, although these treatments slow progression of DN, they do not prevent or reverse it.^[10]^ Present study shows that ACEI/ARB is contraindicated for patients with severe renal impairment and have a set of serious side effects.^[11]^ Although the combination treatment with Chinese medicine and conventional western medicine is considered an effective approach to many conditions, herbal preparations are being preferred as assist therapy, because of their multiple targeted drug actions.^[12,13]^

In recent years, Chinese herbal medicine (CHM) has been widely used in the treatment of diabetes and its complications, it has many advantages over the conventional medical approaches in the prevention of diabetic complications.^[14,15]^ Traditional Chinese medicine (TCM) plays an important role in the early DN of TCM. It has the characteristics of multichannel and multitarget. It can improve the curative effect of routine western medicine treatment, improve lipid metabolism, reduce blood viscosity, improve hemorheology and renal hemodynamics, reduce inflammatory reaction, inhibit the excretion of proteinuria, delay the damage of renal function and protect renal function. A great amount of randomized controlled trials (RCTs) have suggested that CHM alone or combined with ACEI/ARB has therapeutic potential in the treatment of DN can improve reducing urinary albumin excretion, ameliorating proteinuria, and symptoms.^[16]^ However, there is still lack of relative systematic evidence-based medical evidence about CHM in treating patients with early DN currently. Therefore, in this review, we will evaluate the efficacy and safety of traditional CHM (TCHM) in treating early DN in recent 10 years, which aim to provide sufficient evidence for its clinical application.

## 2 Methods

### 2.1 Inclusion criteria

This study will include all the RCTs that relate to CHM therapy in treating early DN. For the included trials, the investigators need to precisely report the stochastic methods, CHM treatment details and parameters, diagnostic criteria, and efficacy evaluation they based on. No limitation to whether it is published or not. The experiment is limited to humans. Language is limited to Chinese and English. Participants who were definitely diagnosed with early DN would be included, but except for patients with other serious disease. There will be no limitation about sex, ages, and other factors. The cases which relate to prostatic hyperplasia, prostate cancer, or other prostate-related diseases would be excluded. The intervention included both prescription and CHMs. The control intervention included simple western medicine, such as placebo or ACEI/ARB. Hypoglycemic therapy was used as a cointervention in both of the arms, including oral hypoglycemic drugs, insulin, and exercise, or did not get any treatment as a blank control would be adopted.

### 2.2 Exclusion criteria

This study will be excluded as follows: literature are nonclinical treatment studies; clinical studies with other diseases; unclear indicators of efficacy; incorrect randomized methods; and studies are literature review or repeated publication.

### 2.3 Outcomes

#### 2.3.1 Primary outcomes

The primary outcome included 24-hour urine protein quantitation, urinary albumin excretion rate, fasting blood glucose, and glycosylated hemoglobin.

#### 2.3.2 The second outcome measure

The 2nd outcome measure is based on TCM syndrome evaluation criteria.

Healing: the clinical symptoms and signs of TCM disappear or almost disappear, and the syndrome score is reduced by ≥90%; urine protein excretion rate, creatinine clearance rate all returned to normal.

Significant effect: the clinical symptoms and signs of TCM are obviously improved, and the syndrome score is reduced by ≥60%, <90%; urine protein excretion rate decreased ≥50%, <70%; creatinine clearance was normal.

Effective: Chinese medicine clinical symptoms and signs have improved, syndrome scores decreased by <60%, but ≥30%; urinary protein excretion rate decreased by ≥20%, <50%; creatinine clearance was normal.

Invalid: the clinical symptoms and signs of TCM were not improved, even worse, and the syndrome score was reduced by <30%.

Integral variation formula (nimodipine method: [(pretreatment score – posttreatment score) ÷ pretreatment score] × 100%.

### 2.4 Study search

We will search in 3 English database including PubMed, Embase, and Cochrane Library Central Register of Controlled Trials and 4 Chinese databases including China National Knowledge Infra structure (CNKI) database, Wan fang Data Knowledge Service Platform, the VIP information resource integration service platform (VIP), and China Biology Medicine Disc (Sino Med) with a language limitation of English and Chinese. Search for clinical research literature on CHM treatment of DN published in domestic and foreign biomedical journals from 2010 to February 2020. Based on the standards of the Cochrane Collaboration Workbook of the International Evidence-Based Medicine Center, a manual and computer-based method will be used to conduct related literature searches. The search terms used will be as follows: Chinese medicine, traditional Chinese medicine, Proprietary Chinese medicine, Chinese herb medicine, early diabetic nephropathy, early-stage diabetic nephropathy, early aged diabetic nephropathy, randomized controlled trial, controlled clinical trial. A sample search strategy for PubMed is shown in supplementary Table 1.

### 2.5 Study selection

**Figure 1:**
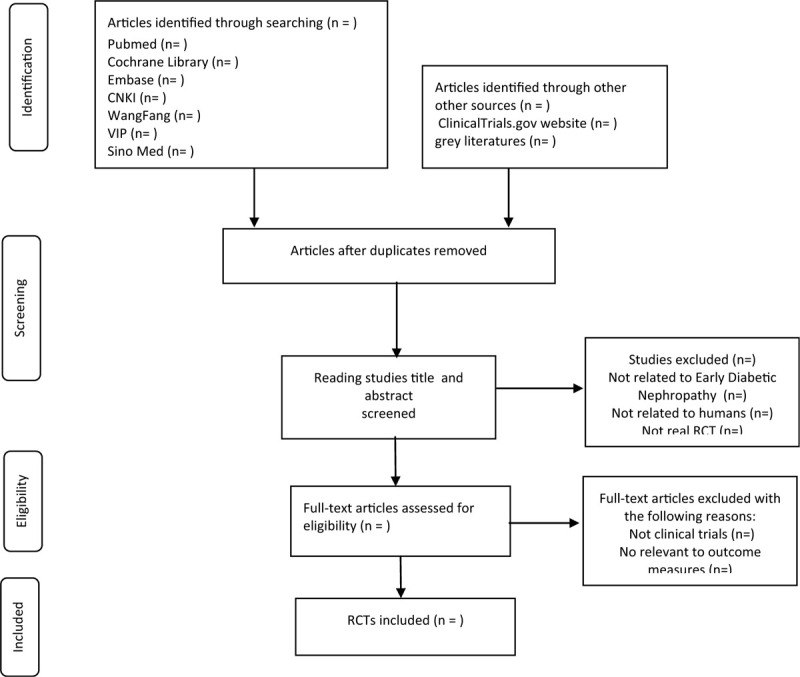
Flow diagram of study selection. CNKI = China National Knowledge Infrastructure, RCT = randomized controlled trial, VIP = the VIP information resource integration service platform.

Two investigators used EndnoteX8 software to conduct a preliminary assessment of the title and abstract of each document in the database based on the established inclusion and exclusion criteria in the study to select eligible studies. Those articles that meet the criteria will be further determined for inclusion by reading the full text. Any differences in screening that occurred during the screening study would be discussed in order to get consensus, if it still cannot be resolved, then the 3rd author would be intervened. Reviewers will document the reasons for exclusion and the whole eligible process will be shown in the PRISMA flow diagram (Fig. 1).

### 2.6 Data extraction

Two investigators independently extracted information from the included literature. The extracted content includes year of publication, research design, random hiding and blinding, basic information of the included cases, intervention methods, observation indicators, and test results of the treatment group and the control group. The extracted literature data will be filled in a unified data statistics table. If there is not enough data in a study, we will contact the corresponding author for more detailed data. If the methodologic details are not told in papers, we will contact for more explanation.

### 2.7 Assessment of risk of bias in included studies

Two investigators will independently evaluate the methodologic quality of the included literature by using the Cochrane Collaboration's ROB tool which includes 7 items: random sequence generation (selection bias), allocation concealment (selection bias), blinding of participants and personnel (performance bias), blinding of outcome assessment (detection bias) incomplete outcome data (attrition bias), selective reporting (reporting bias), other bias. According to the relevant standards in the Cochrane Intervention System Evaluation Manual, it will be divided into low risk, high risk, and unclear.

### 2.8 Data analysis

We will use the normalized mean difference with a confidence interval of 95% express the numerical variable. Chi-squared test (test level a = 0.1) will be used to test the heterogeneity of each pairwise comparison. If there is no heterogeneity, a fixed effect model will be used. If there is significant heterogeneity in a group of studies, we will explore the reasons for the existence of heterogeneity from various aspects such as the characteristics of the subjects and the degree of variation of the interventions. Sensitivity analysis or meta-regression and subgroup analysis to explore possible sources of heterogeneity when necessary.

### 2.9 Assessment of heterogeneity

We will explore the reasons for the existence of heterogeneity from various aspects such as the characteristics of the subjects and the degree of variation of the interventions, if there is significant heterogeneity in a group of studies. Sensitivity analysis or subgroup analysis is performed as necessary to explain heterogeneity. Subgroup analyses were carried out by region, sample size, and types of DN for there was significant heterogeneity across the included studies.

### 2.10 Sensitivity analysis

We will conduct a sensitivity analysis for the outcomes to investigate the stability of the results. We will exclude each study 1 by 1 which is included in the analysis, and then reanalyze and pooled the data and compare the difference between the reobtained effects and the original effects. In this way, we will be able to assess the impact of individual studies on the overall results and whether the results are strong.

### 2.11 Publication bias assessment

The funnel plot were drawn and analyzed by using RevMan 5.3 software, it was used to analyze potential publication bias, and Egger test will be conducted for statistical investigation.

### 2.12 Grading the quality of evidence

The quality of evidence for the main outcomes will also be assessed with the GRADE approach. In this table, the evaluation will be shown from 5 domains: certainty assessment, number of patients, effect, certainty, and importance. In the GRADE system, the quality of evidence can be defined as “very low,” “low,” “moderate,” or “high” judgment.

### 2.13 Patient and public involvement

Patient and public were not involved in this study.

### 2.14 Ethics and dissemination

Ethical approval is not needed for this meta-analysis. Our study comprehensively evaluates the existing research evidence of traditional CHM and is bound to provide evidence-based medical support for clinical workers. The results of our research will be published at a peer reviewed journal.

## 3 Discussion

The DN is one of a serious complication associated with diabetes mellitus which can cause ESRD. The major treatment to slow the progression of diabetic kidney disease (DKD) is use the ACEI medications when the proteinuria was appeared, and effectively control and management of high blood pressure and blood sugar levels are also essential for DKD treatment.^[5]^ Modern medicine generally adopts comprehensive treatment for DN, including health education, diet and exercise management, control of blood sugar, regulation of blood pressure and blood lipids, prevention of complications and other symptomatic treatment, later use of blood purification, kidney transplantation, and other treatment, but so far there is no effective western medicine can prevent the natural process of kidney function damage in DN patients. However, lots of adverse effects of ACEIs and ARBs, such as hyperkalemia, rhinitis, acute kidney injury, persistent cough, and angio-edema, exclude the continuous use of this therapy in many cases.^[17–20]^ Thus we need more efficacious therapy to treat patients with DN. Clinical practice shows that the combination of traditional Chinese and western medicine treatment DN can effectively improve clinical symptoms, protect renal function, and show obvious advantages in DN early prevention and treatment. Study shows that TCHM has been long used to treat complications of DM, including DKD, and has a great ability to improve patients’ quality of life. Many in vitro and animal studies have demonstrated the biologic activity and therapeutic effects of TCHM.^[17]^

In recent years, CHM has been widely used in the treatment of diabetes and its complications. Some studies contribute the protection of TCM to their anti-oxidative and anti-inflammatory properties. For example, there are studies shown that LWDH and Ginkgo biloba may attenuate deterioration of albuminuria in type 2 diabetes patients. Their results suggest that TCM is a promising option of protective agents for early stage of DN.^[21]^ There are studies also suggest that LDP could obviously inhibit the activity of EAR in patients with early DN, improve various indexes of DN, so as to be helpful for its treatment.^[22]^ Besides, study shown that ZSTL is superior to benazepril at improving the metabolic and renal functioning in patients with early stage DN, in part, by modifying ANP, ET-1, and VEGF.^[23]^ And there is a study aim to explore the mechanism of acanthopanax senticosus (AS) on DN patients, they find that the levels of urinary albumin excretion, plasma and urinary ET lowered significantly (*P* < 0.01) in the AS groups, and the protective effect of AS injection on DN is probably correlated with its inhibition on ET synthesis in kidney.^[24]^ Study also shown that Shenkangwan can provide renal protection against DN in rats and alleviate the structural and functional damages of podocytes possibly by reducing desmin expression and increasing podocin expression in the podocytes.^[25]^ Many studies found that traditional CHM had a positive activity in treatment of early DN patients.

At present, there are few systematic reviews on traditional CHM for the treatment of early stage of DN. Therefore, this study will provide evidence of the effectiveness and safety of traditional CHM in treating patients with early DN in recent 10 years, aims to comprehensively analyze the clinical efficacy of TCHM in the treatment of early DN.

## Author contributions

XMY conceived the idea and XMY, CLH designed the study. XMY and SJW reviewed scoping searches and contributed to the methodologic development of the protocol. XMY and CLH drafted the initial manuscript and all the authors (SJW, QC) revised the manuscript. All the authors have given approval of publishing. QC is the review guarantor.

## Supplementary Material

Supplemental Digital Content
