# Supplementary material for: Clinical efficacy and safety of Chinese herbal medicine for the treatment of patients with early diabetic nephropathy: A protocol for systematic review and meta-analysis
Source: Medicine (Baltimore). 2020 Jul 17;99(29):e20678. doi: 10.1097/MD.0000000000020678 (PMC7373501; doi:10.1097/MD.0000000000020678)
Supplement: Supplemental Digital Content [file medi-99-e20678-s001.docx]

**Table 1 The complete PubMed search strategy is summarized in**

| **Number** | **Search terms** |
| --- | --- |
| 1 | Chinese medicine |
| 2 | Traditional Chinese medicine.ti, ab. |
| 3 | Proprietary Chinese medicine. ti, ab. |
| 4 | Chinese herbal medicine. ti, ab. |
| 5 | Or 1-4 |
| 6 | Early Diabetic nephropathy |
| 7 | Early-stage Diabetic nephropathy .ti,ab |
| 8 | early aged diabetic nephropathy |
| 9 | Or 6-8 |
| 10 | Randomized controlled trial. pt. |
| 11 | Controlled clinical trial. pt. |
| 12 | Randomized. ab. |
| 13 | Placebo. ab. |
| 14 | Randomly. ab. |
| 15 | random allocation. ab. |
| 16 | single blind. ab. |
| 17 | double blind. ab. |
| 18 | Trial. ab. |
| 19 | Or 10-18 |
| 20 | Exp animals/not humans. sh. |
| 21 | 19 not 20 |
| 22 | 5 and 9 and 21 |
